# Food insecurity and levels of marginalization: food accessibility, consumption and concern in Mexico

**DOI:** 10.1186/s12939-023-01977-5

**Published:** 2023-09-04

**Authors:** Oscar A. Martínez-Martínez, Karol Gil-Vasquez, María Beatriz Romero-González

**Affiliations:** 1 Universidad Iberoamericana. Department of Social and Political Sciences, Prolongación Paseo de la Reforma 880, Álvaro Obregón, Lomas de Santa Fe, México City, 01219 México; 2 Boston University, 264 Bay State Rd, Boston, MA 02215 USA

**Keywords:** Food Insecurity, Marginalization, Concern, Lack of food, México

## Abstract

**Background:**

Food insecurity continues to be a problem throughout the world. When estimating food insecurity, few studies analyze the contexts where the phenomenon takes place. By bearing in mind levels of marginalization in four states of Mexico, this paper answers two questions: (I) What problems are experienced with access to food, and how these difficulties affect the amount of food consumed in households? and (II) How do households experience the concern of running out of food?

**Methods:**

Our qualitative study draws data from urban and semi-urban areas of four Mexican states: Mexico City, Tamaulipas, the State of Mexico, and Oaxaca. Each state presents different levels of well-being. The study’s participants are selected using the snowball method. Eligibility criteria are based on demographic characteristics such as education, age, and gender. A thematic analytical approach is conducted to analyze collected data from a total of 212 semi-structured interviews.

**Results:**

The study’s findings indicate that concern of food scarcity is a generalized feeling among participants across different levels of marginalization. Individuals with stable jobs living in contexts of low levels of marginalization experience worriedness when their budgets tightened before the end of the payday, a bi-weekly payment format, named the *quincena* in México. This psychological state of mind changes through the payday cycle, a period when the direct relationship between food accessibility and consumption weakens. In response, individuals develop strategies to cope with the uncertainty of experiencing food insecurity, such as rationing food portions and/or hoarding food supplies. Even when food accessibility exists, interviewees identify insufficient income as the primary issue in contexts of low and very low levels of marginalization.

**Conclusions:**

Conclusive remarks drawn from our analysis underline the importance of the context of marginalization in influencing households’ experiences with food insecurity. At the *quincena’s* end, food insecurity increases, even in contexts of very low marginalization. Our study calls for rethinking the scales employed to measure food insecurity, specifically the questions related to fear of food scarcity. Coping strategies are implemented by surveyed individuals to resolve issues and repercussions that emerge from experiencing food insecurity differ by context of marginalization.

## Background

Food provides the basic physical and psychological means to obtain and maintain well-being. The concept of wellbeing integrates objective factors such as health, income, and food as well as subjective factors such as perceptions of a satisfactory lifestyle, built environment, and social capital [1]. Within the comprehensive interpretation of well-being, food has a primary role to play since food consumption has a direct impact on objective factors like health, and subjective factors like feeling satisfied with one’s life [2]. Hence, adopting strategies to modify the quality and quantity of food consumed at home such as reducing portions, skipping meals or being unable to consume a varied diet generates food insecurity. Food insecurity is defined as the limited or uncertain availability of nutritionally adequate and safe foods; or the limited and uncertain ability to acquire adequate food in socially acceptable [3] and culturally appropriate ways [4, 5]. When food insecurity is severe, people experience hunger. Hunger is an extreme condition under which food supplies are entirely scarce and individuals do not eat for a day or more [6].

Approximately 2.3 billion people lack access to food [7]. Recently, food insecurity has captured global attention after unprecedented inflation and severe disruptions in global supply chains are aggravated by the COVID-19 pandemic and War in Ukraine. Notwithstanding, no region in the world, from both the Global North and the Global South, has entirely resolved the issue of food insecurity [8]. Food insecurity presents serious challenges to achieving the UN Sustainable Development Goals 2 (SDG2), which foresee ‘a world without hunger and malnutrition.’ Related to food insecurity is food sovereignty. Food sovereignty is defined as the ability to develop a food system in which the people who produce, distribute, and consume food also control the mechanisms and policies of food production and distribution. Food insecurity as well as food sovereignty are serious issues in México, a country that has seen the right to define its agricultural and food policy threatened for years [9, 10].

Food insecurity is measured through different instruments including the Food Security Scale of Latin America and the Caribbean (ELCSA), the FAO Food Insecurity Experience Scale (FIES) and the Mexican Food Insecurity Scale (EMSA). EMSA shares the purpose of the FIES and ELCSA within the Mexican context. These instruments typically include the following dimensions: (a) the concern about food scarcity in the household; (b) the quantity and quality of food; (c) the omission of any of the meals by household members; (d) the sensation of hunger by household members; and (e) the suspension of any of the meals in the household due to lack of money and/or other resources. Undoubtedly, scales to measure food insecurity incorporate solid dimensions of analysis that guarantee validity and reliability [6]. In addition to providing an affordable way to measure food insecurity, scales are more accessible compared to other instruments. Nonetheless, scales also present limitations. They are unable to measure food insecurity among children, pregnant women, and individuals with specific characteristics [11, 12]. The imprecise measurement of food insecurity posits a serious obstacle to designing an adequate policy to eradicate it.

People who suffer from food insecurity see their living standards decline [13], their physical and mental conditions, specifically. Individuals who confront food scarcity at some point in their lives tend to suffer from insomnia, diabetes, hypertension [14, 15] and obesity due to consumption of inexpensive high-calorie food [16, 17]. Older adults are more likely to suffer from arthritis, joint pain, and physical disabilities related to being overweight [18]. Equally, food insecurity drains individuals’ mental energy over the concern of not having enough food to satisfy biological needs [14], triggering depression [19].

According to the National Council of Evaluation (CONEVAL – in Spanish), only 57.8% of the Mexican population can secure food for consumption, while 42.2% (around 53.5 million people) experience food insecurity [20]. Approximately 8.1% of the population (close to 10.2 million people) encounter severe levels of food insecurity. This share is represented by individuals who are left behind with no food —unable to eat for a day or more. Experiencing severe levels of food insecurity is a deplorable inhumane condition that sets the stage for undesirable experiences [3]. Between 2016 and 2020, according to CONEVAL, food insecurity increases before the COVID-19 pandemic. The spike on this rubric is directly connected to the country’s unfavorable macroeconomic conditions that first impact Mexican households. Detrimental factors that impair individuals from meeting their food demands include higher unemployment and poverty rates, booming inequality, and inflation [21].

Food insecurity has a different impact on urban, semi-urban, and rural areas [22]. Subjected to residential context, households allocate different amounts of resources in transportation to obtain food. Rural locations spend more on transportation to buy groceries while neighborhoods located in urban areas have automatic access to supermarkets or convenient stores through transiting urban infrastructure: walkways, avenues, and highways [23]. Due to geographic isolation, households located on the outskirts of cities encounter more obstacles to accessing food. In turn, food becomes more expensive given the incurred cost in transportation to obtain it. Thus, levels of marginalization generate distinct degrees of food insecurity [24]. Semi-urban areas are estimated to face food insecurity more often due to their disconnection with urban centers. A negative circumstance that is accentuated by the economic conditions that characterize these contexts [25].

Furthermore, food insecurity is aggravated with the presence of children and older adults [2]. When a household experiences food scarcity, specific household members are prioritized over others. This strategy is incorporated into family dynamics to confront food insecurity [26]. For this reason, households implement different coping strategies, such as sharing smaller portions of food with all family members, including those who live in a different household, but are affiliated with the extended family [27]. Other strategies include borrowing money or purchasing food with credit, changing family diets, and/or starving [28]. Similarly, individuals stop buying food and/or items that are not necessities; eat expired or spoiled foods [29]; and buy fruits and/or vegetables of lower quality from street vendors [30]. Families also search for donations or utilize programs and initiatives created by religious and civic organizations, often supported by the government, to satisfy their daily meals [31, 32]. More severe strategies to cope with food insecurity are adopted as food access is more restrictive [33].

Regional contexts and other socioeconomic factors also determine food insecurity. Consequently, analyzing how experiences of food insecurity are shaped by diverse contexts of marginalization is of the utmost importance to advance the study of food insecurity. Our analysis addresses the following two questions: (I) What problems are experienced with food access, and how this affects the amount of food consumed in households? (II) How do households experience the concern of running out of food? We argue that a study on contemporary food insecurity demands an insightful analysis to design competent public policies. Our study accounts for the context where food insecurity is generated and regenerated. By considering levels of marginalization, findings from this study indicate that food insecurity breeds diverse experiences. In this sense, the gap this effort aims to close is rethinking how food insecurity is measured by delving into how the context of marginalization can affect food accessibility, food consumption, and the concern of food scarcity. The study’s ultimate objective aims at rethinking current scales utilized to measure food insecurity.

## Methods

### Measurements

The study accounts for four central measures: food access, the amount of food consumed, concern about food scarcity, and levels of marginalization. The first three measures build upon FAO [34]’s conceptualization of food insecurity, as well as the analytical frameworks provided by the ELCSA, EMSA, and FIES scales. Resting on these parameters, access to food is understood as the availability of finding nutritionally adequate food in socially acceptable and culturally appropriate ways in the context where individuals live. The amount of food consumed by households refers to the portion ingested by household members in terms of quality and quantity, which is reflected in its variety, portions, and the number of times meals are skipped. Concern of not having enough food refers to the uncertainty felt from food scarcity.

The Municipal Marginalization Index (IMM) is a scale created by *the Consejo Nacional de Población* (Mexico’s National Population Council) to gauge levels of marginalization at the territorial level. The IMM considers states and municipalities as units of analysis [35] and encompasses four indicators: (1) education or illiteracy: percentage of the population with very low levels of education (incomplete elementary school); (2) housing services: percentage of households that lack plumbing, sanitary services, electricity, water pipes, paved floors and present overcrowding; (3) population distribution: percentage of municipalities with less than 5,000 inhabitants; (4) income: percentage of workers that earn up to two times the minimum wage. The IMM segments levels of marginalization into five categories: very high, high, medium, low, and very low. Municipalities with very high levels of marginalization are the most vulnerable, while those with very low marginalization present the highest standards of living conditions.

### Study sample and data collection procedures

The study collects data in México City (high), Tamaulipas (medium), the State of México (low), and Oaxaca (very low), each of these entities represents different levels of social well-being [1]. In each entity, urban and semi-urban municipalities are selected (see Table 1). The sample of municipalities is heterogeneous based on levels of marginalization and population’s size [36]. The research incorporates 60 municipalities, distributed as follows: México City (16), State of México (28), Oaxaca (10), and Tamaulipas (6). An attempt has been made to cover different levels of marginalization. However, according to CONAPO data [35] not all levels of marginalization are represented by the places where the research is conducted. Still, whenever the selected municipalities present similar levels of marginalization, for convenience, the municipality with the easiest access was chosen. The research’s primary objective is to include municipalities that distinguish themselves from each other. Based on findings from other qualitative studies, differentiation strengths information’s quality and guarantees the saturation of categories [37].

Semi-structured interviews have been administered to 212 individuals. The sample allows for building up a total of 146 semi-structure interviews that were conducted in municipalities with very low levels of marginalization. Forty-seven (47) semi-structured interviews originate from localities with low levels of marginalization. This sample is followed by 7 semi-structure interviews from localities with medium levels of marginalization, 9 from municipalities with high levels of marginalization, and 3 from very high levels of marginalization. Given the limited number of interviews conducted in high and very high levels of marginalization, the two categories are pooled into one analytical section. Details on semi-structure interviews by contexts and levels of marginalization are described in Table 1.

For each state, a group of residents has been recruited and trained in data collection. During the data collection process, interviewers strive their best to communicate as accurate as possible the meaning of the information shared by participants, taking advantage of their familiarity with cultural nuances specific to the state where the data was collected. Interviewers have received training in ethical standards and data management. For recruitment efforts, personal networks assist researchers in reaching out to potential participants. In each municipality, the snowball technique is employed to recruit participants with adequate profiles for this research. Although the selection process rests upon a non-probabilistic convenience sampling, demographic characteristics such as age, gender, and educational level, are considered to build a sample with a heterogenous profile. All semi-structured interviews are conducted in Spanish. Each interview lasts an average of 45 min.

The semi-structured interview is divided into the following sections: (1) Sociodemographic characteristics; (2) Food access; (3) Food consumption; (4) Concern about food scarcity. Based on the framework of a semi-structured interview, interviewers formulated additional questions when deemed appropriate to delve into how food insecurity is experienced. The study has been approved by the Ethics Committee of the Universidad Iberoamericana-Ciudad de México. Participants have signed an informed consent form before conducting and recording each interview. The questionnaire format is included in Table 2.

**Table 1:** Interviews by IMM

Research sites	Type of municipality	IMM	Number of interviews
México City	Urban: 100%	Very low	70
		Low	8
Tamaulipas	Urban: 88.6% Semi-urban:11.4%	Very low	39
		Low	5
State of Mexico	Urban: 59.6% Semi-urban: 40.4%	Very low	21
		Low	8
		Medium	6
		Very high and high	12
Oaxaca	Urban: 90.7% Semi-urban: 9.3%	Very low	16
		Low	26
		Medium	1

Source: Author’s elaboration

### Data analysis

Each interview has been transcribed utilizing a word processor. To their best capabilities, authors respect the participants’ voices and expressions when performing translations into English as verbatim as possible. Each transcript has been transferred to the Atlas T.I. program. To analyze interviews’ transcripts, researchers employ a thematic analysis approach which incorporates a method for identifying, analyzing, and reporting patterns or themes from qualitative data [18]. The code book is determined based on our review of literature on food insecurity (see Table 2). Authors utilize deductive coding to create a link between theory and observations [38]. During the research project, authors have met on several occasions to discuss and analyze data. Research meetings are triangulated with findings and interpretations from the literature review.

**Table 2:** Codes Used in Analysis

Dimension	Codes	Questions
Food Insecurity	Food access in the household	How difficult is it to get food in your neighborhood, town, or municipality? Where do you usually buy food? Why?
	Food consumption in the household: a) Food quality and quantity b) Variety of foods c) Portion size d) Skipping meals or not eating	Is the food you eat at home enough for the whole family? Have you ever had to buy less food or lower the quality of food? Why? What would it mean for you to have varied nutrition? Have you been able to maintain varied nutrition in your home? Why? Did you or any member of the household skip any of the meals that you are used to eat? Why did this happen? At any time, did you stop eating for a whole day? Why did this happen?
	Concern about food scarcity	Have you been concerned about running out of groceries, and you won’t be able to buy more? How often does this happen? What do you think about it?

Source: Author’s elaboration

## Results

### Sample - demographic characteristics

Table 3 presents the sample’s demographic characteristics. The average age and gender distribution among participants is similar at all levels of marginalization. Regarding education, data from very low and low levels of IMM exhibits the largest percentage of participants who graduated with university and/or post-graduate degrees. A variety of occupations are included in the sample. Participants who report being formally employed are grouped into categories that account for different economic sectors. Another share of participants is unemployed, working for the informal sector and/or providing full-time care for family members. Interviewees’ characteristics are connected to the context where they live. Municipalities with low and very low marginalization conglomerate the highest number of graduates from institutions of higher education, skilled workers with benefits, and self-employed individuals (free-lancers) in white-collar occupations. In contexts of high and very high levels of marginalization, the level of education of participants is low. The highest concentration of informal occupations and unpaid jobs is found in these last categories.

**Table 3:** Characteristics by Marginalization’s Levels

Level of Marginalization	Average age	% Gender	% Educational Level	Type of job
Very Low	44	Male: 47.3 Female: 52.7	No Education: 2.7 Elementary: 11.6 Junior High School: 19.2 High School: 31.5 College: 23.3 Post-Graduate:11.7	Tertiary sector: 72% Unpaid labor: 10% Secondary sector: 5% Informal work: 5% Retired: 5% Unemployment: 3%
Low	45.5	Male: 53.2 Female: 46.8	No Education: 14.9 Elementary: 14.9 Junior High School: 14.9 High School: 31.9 College: 21.3 Post-Graduate: 2.1	Tertiary sector: 52% Secondary sector: 15% Unpaid work: 9% Informal work: 9% Unemployment: 9% Retired: 6%
Medium	43	Male: 57.1 Female: 42.9	No Education: 14.3 Elementary: 28.6 Junior High School: 14.3 High School: 28.6 College: 14.2	Tertiary sector: 43% Informal work: 28% Unpaid work: 15% Retired: 14%
Very High and High	47	Male: 41.7 Female: 58.3	No Education: 8.3 Elementary: 33.3 Junior High School: 16.7 High School: 31.3 College: 10.4	Tertiary sector: 66% Informal work: 25% Retired: 9%

Source: Author’s elaboration

### Food access and consumption

#### Very low levels of marginalization

Based on the IMM, households with very low levels of marginalization display the highest living standards of the sample. 93% of the interviewees have no issues with accessibility. To supply food demands, participants visit near convenient venues, including supermarkets and *tianguis*. Visiting a *tianguis* is an old practice in Mexico. Generally, a *tianguis* is a market that offers fresh and locally produced vegetables and fruits at affordable prices. Among participants, *tianguis* stands as the preferable option in terms of food quality and price. For small purchases, *tianguis* is seen as a convenient venue that keeps in mind the limited budget of an average consumer. Factors that influence selecting the commercial venue to purchase food include the following: location, amount of disposable income, and quality of fruits and vegetables:

*“We prefer the tianguis because of their fresh products, besides, [it] ends up being a little bit cheaper…[tianguis] is better in terms of quality and price almost all the time… When there are sales of something in the supermarket we buy [in this venue], but at times…fruits are no tripe enough, even though [they are] cheaper. You end up throwing them away.” (31-year-old woman)*

*“[We buy] in the tianguis because fruits and vegetables are fresher and cost less and are of higher quality. [We buy sometimes] in the supermarkets because sometimes buying a little bit of more quantity helps you in planning until the next quincena.” (40-year-old woman)*

Regarding consumption, 71% of the participants have enough food to satisfy the household’s needs. In this respect, 66% of respondents can maintain—what they considered to be—a variety diet, particularly when medical recommendations are given and associated with the presence of a(n) ill family member(s) in the household. Nonetheless, some of these interviewees recognize the negative impact their low earnings have on the quality and quantity of food purchases. Food portions are insufficient to feed all family members.

*“Well yes, lately we’ve tried to increase food variety because before, well no, I believe that all these [factors] influence to be in good health. And now, when one is sick, now, one tries to eat better.” (51-year-old male)*

*“Yes, thanks God, now we do plan for the week, what’s needed for [food] consumption over the week.” (45-year-old woman)*

*“Mh well […] sometimes it’s like this, because at times it’s not enough now. Yes, the money, but lately, we’ve prevented this [shortage]. Well, thanks God we are better established, that is, better programmed on what needs to be purchased in the weekends for the rest of the week. Afterwards, we buy [food] as needed.” (56-year-old male)*

#### Low levels of marginalization

In contexts of low levels of marginalization, 73% of the interviewees face no difficulties purchasing food in their neighborhoods. The main criterion for food selection is a combination of price, location, and quality. Despite having no difficulties with food availability, low-income levels remain the primary issue that limits the interviewees’ ability to secure food for consumption within a context of high levels of IMM:

*“No difficult, it is not difficult, because there is food everywhere, the problem one has is no money, that’s the problem.” (43-year-old male).*

*“Well, to save [you buy] in the supermarkets […] one only visits the [convenient] store only because you are in a hurry. In big establishments [supermarkets], we don’t save much but we can buy more things.” (40-year-old woman).*

In this category, almost all interviewees (94%) report having enough food to meet household needs. Even though some participants face difficulties, they have not skipped any of the three daily meals. Nonetheless, some of these participants must reduce food consumption to secure supplies for the entire family. A three-meal pattern is an eating habit that appears in contemporary societies to replace de-structured forms that involved greater breaks between food intakes, snacking and non-ritualized settings of food consumption [39]. Such pattern is traditionally served in Mexican households to this day. Not being able to purchase enough groceries causes a feeling of concern that pressures individuals to ration food, giving priority to the youngest family members. In this case, a sense of sacrifice on behalf of own’s children is taken for granted, generating a food shortage that takes a toll on the breadwinners:

*“Yes, we always plan for all of us to eat. Something our parents have taught us, our grandparents, is that always all [family members] need to eat and the parents are the last ones to be fed, right? Parents sacrifice themselves for their children. But well, we are not expecting to sacrifice ourselves. We hope that all [family members] are satisfied.” (55-year-old male)*

Despite having no issues with accessibility, individuals are unable to purchase food of high quality due to their low earnings. Generalized low levels of income across categories of marginalization underline a distinction between issues related to accessibility versus consumption. Living in a neighborhood that grants access to food does not necessarily guarantee that required levels of food consumption to be in good health will be met:

*“Sometimes we look for the cheapest food, to make ends meet.” (35-year-old woman)*

*“Well yes, because there is sometimes well…if we don’t have enough, we must figure it out…if I’m going to [usually] eat two or three tortillas, well, I only eat one [tortilla] so there are enough [tortillas] left for my children.” (39-year-old woman)*

*“We don’t have enough to buy everything at once […] if you buy chicken, then you don’t buy milk. If you buy milk, you don’t buy, well, fruits, or buy fruits and no milk […] I mean there is always something lacking.” (37-year-old woman).*

#### Medium levels of marginalization

Resembling descriptions from the previous category, localities characterized by medium levels of marginalization experience no difficulties with accessibility. In these neighborhoods, enough venues to purchase food such as a *tianguis* or convenient stores can be found nearby. Once again, the low earnings are still reported as the primordial limitation to buying enough food.

*“I believe that right now, no, it’s not difficult to get food…the difficulty is to have the economic resources to be able to…well, to obtain it. And well, I believe that this is the most difficult.” (45-year-old male).*

In this category, *tianguis*, convenient stores, and supermarkets comprise places where interviewees purchase food, setting as main criteria for food selection: price, quality, and variety.

*“At different stands of the tianguis, for example, at the butchery that we have here, the fruit store. Also, there is Lores (a self-service store). We also go to Tuxtepec, to the big stands that they have. Sometimes we take advantage of going to Tuxtepec, and just get there, and calculate where food is a little cheaper.” (60-year-old male).*

*“The other advantage that we have is that we live only ten minutes away from the city of Huajuapan de León. There, one can find practically everything.” (40-year-old woman).*

Regarding food consumption, 85% of surveyed individuals indicate that no family member has gone a day without eating food. However, the same interviewees must skip one of the three meals of the day in addition to reducing food quantity and quality. These participants must prioritize the payment of other expenses in the household (such as the imbursement of utilities) to make ends meet all the way to the *quincena’s* end. Other expenditures take prominence in the household’s budget. Consequently, resources allocated for food consumption and purchases of high-quality food are reduced:

*“Mh no, thanks God, at least beans, we’ve had food to be able to eat the three meals of the day.” (30-year-old male)*

*“Here sincerely well […] for lack of employment […] sometimes we’ve not had enough economic liquidity [financial stability] to be able to eat three times a day. But my daughters do, I try my best so they can always have three meals per day. At least we always eat beans and eggs.” (42-year-old woman)*

#### Very high and high levels of marginalization

Interviewees at very high and high levels of marginalization live in the most vulnerable contexts. Even though these areas are considered semi-urban localities, they are still located at a far distance from urban centers. Among participants under this category, food accessibility issues are present. The long commute from homes to urban centers creates a natural barrier to access food. Experiencing issues with transportation also enlarges the distance and increases the time to gain accessibility. Issues with transportation—such as not owning a vehicle or having to take several rides to reach the closest urban center—impose a serious inconvenience to approach centers of food distribution. In this case, participants strive to find areas/neighborhoods where food stores offer variety, higher quality, and lower prices:

*“In Santa Fe, the municipal head, this is where I purchase, every Sunday holds a market. And, well, there are some who purchase two weeks of food supplies, but it depends on one’s budget, also. But to go to Santa Fe, you must also pay the bus fare or find someone to give you a ride, and one only pays gasoline expenses. The trip lasts almost an hour” (31-year-old male).*

*“… When we go to Ixtlahuaca, days before we put some coins aside …for example, the [cooking] oil is a little bit cheaper in Supercompras [convenient store] or Soriana [supermarket].” (57-year-old male).*

Limited consumption of food represents a serious challenge. Purchasing food outside of the neighborhood signifies a higher cost. In addition to low-income levels, the context indirectly reduces the quantity and quality of food to be purchased. As a result, 93% of the interviewees acknowledge the lack of economic resources to afford a variety diet:

*“To be able to feed ourselves with a variety of foods, unfortunately sometimes, one cannot [do so] for the low salary or the high prices of the basic consumption basket. You go and purchase something from the store and spend 100 [Mexican pesos], 200 [Mexican pesos], one does not earn [those monies] in one day.” (41-year-old male).*

*“[…] If there is no money, sometimes only for example you buy, what can I tell you? potatoes and there will be no meat, and there are […] no other things, the cheese, the milk, or something like this. And well, it’s like this under these conditions.” (47-year-old woman).*

The descriptions above are supported by 48% of the interviewees, who after identifying the sharp increase in food prices had to reduce food quantity and quality due to insufficient disposable income. In this category, 80% of the interviewees have had to eliminate one of the three meals of the day:

*“Above all, at the end of the payday, there is not much money, well like I was telling you at the beginning, we must look for substitutes or look for less expensive food.” (32-year-old woman).*

*“It happened at some moment that I was laid off. I was very sad but, did not last long… [During that time,] [w]e stopped having dinners to economize [reduce cost].” (35-year-old woman).*

### Concern about Food Scarcity

#### Very low levels of marginalization

At low levels of marginalization, interviewees prioritize buying sufficient food supplies. Nourishment is counted as one of the primary factors of well-being among participants in this category. The novel finding in this regard is how decreasing income levels negatively impact consumption among individuals, who are presumed to hold the highest standards of living:

*“It’s a little of a difficult situation to set limits and limits more on what relates to food or to stop doing other things to be able to eat […] First and foremost is food, but at times, other factors gain [prominence] and you must stop doing something to cover [these expenses], taking a toll on nourishment.” (56-year-old woman)*

In this category, around 56% of the participants feel concerned about the possibility of running out of food, particularly at the *quincena’s* end. For some interviewees, job instability or precarity becomes a key factor that increases levels of concern in a context often assumed to have individuals with sufficient income to meet food demands:

*“Now, well, when one is working you don’t feel it, because to say something, we get a good job in two or three weeks and there we must move forward while another good job drops on us.” (60-year-old male)*

*“Oh, true that! Because sometimes one says: damned! The money is gone, and I am in need [of food] and the pantry is empty, and I don’t have [money].” (42-year-old woman)*

#### Low levels of marginalization

Mirroring findings from the category described above, interviewees from low levels of marginalization also feel concerned over the possibility of facing food scarcity, particularly individuals with children.

*“Well yes, yes, yes, really, I’ve worried about food. I mean about food supplies, because well, one can handle everything little by little, but well […] my children.” (34-year-old woman)*

This cluster of participants manifests less frequent worries. Nonetheless, worriedness intensifies at the end of the *quincena* or when food demands cannot be met due to lack of money. Their uncertainty relates to participants not having a permanent job that provides a stable source of income. Medical expenses incurred by the household to unexpected illnesses tighten the family budget, restraining individuals’ ability to work. The difficult condition further reduces the low income earned in unstable-part-time jobs. In these case scenarios, food insecurity exacerbates when a breadwinner suffers a sickness-related setback:

*“Obviously, I’m the head of the household and I’ve felt sick and have not had the chance to work, so they deduct from my salary the days that I don’t work (53-year-old male)*

*“At the end of the quincena, [there] is not enough [money] to purchase meat or chicken sometimes, one needs to eat more plain things.” (29-year-old woman)*

*“Yes, well there are things that we cannot buy, for instance, canned tuna or things like that are more expensive. For example, I buy cheddar cheese or double cream cheese, but cheaper, but they would like to have gouda cheese, but this one is more expensive. Well, one would like to have turkey breast, but it costs more, and there are some [turkey breasts] that are very cheap, but don’t taste good […] So, there are things that we cannot buy.” (41-year-old male)*

#### Medium levels of marginalization

In this classification, interviewees express worriedness about food scarcity. Several interviewees pursue strategies to avoid skipping one of the three meals of the day. Once again, not having enough money increases feelings of concern about food scarcity. The level of concern intensifies days before the *quincena*. Lack of money is the reason why some participants accumulate non-perishable edibles to guarantee supplies for the family. With the purpose of stretching out already depleted supplies, food is accumulated until the next *quincena’s* payment:

*“Yes, we’ve been worried because when we’ve nothing in the pantry and there are still days left before the pay day[…] That, generates a lot of concern.” (57-year-old woman)*

*“When one has money all the month’s pantry is purchased—the non-perishable food staples—and then, one only buys perishable items daily.” (67-year-old male)*

#### Very high and high levels of marginalization

In this category, 67% of the interviewees identify feelings of concern over the possibility of running out of food or not being able to satisfy their needs or those of family members, particularly when children and/or older adults are present in the household. Some interviewees have confronted food scarcity in the past. This experience severely impacts their perceptions of uncertainty and concern about running out of food again. In this situation, individuals struggle to ensure food supplies, granting priority to buying food above paying other household expenses:

*“Well […] no, it feels really bad, isn’t? One gets desperate, you can say at the beginning, if someone gives us a taco or something like that, one says: well no, I don’t want to be in that situation—well, it does feel ugly, one reacts and says: well, work hard, hustle it!—and there is no way out but that’s it! Thanks God! here we go, our economy began to improve.” (65-year-old woman)*

A significant share of interviewees in this category identifies two key factors of concern: increasing food prices (100% of the participants) and a decline in agricultural production (67% of the participants):

*“Feeding has always been my priority […] then, I believe that, well, is the last item that I would cut back from the budget even though everything is more and more expensive overtime.” (50-year-old male)*

*“Ungracefully, the farmer is no longer harvesting the land […] and it’s going to be a fucking moment when we will have no food. And even, if we have the fucking money to buy food, there is not going to be food.” (31-year-old male)*

## Discussion

A plethora of factors generate food insecurity including mounting poverty, unemployment, and inflation [21]. Based on the study’s findings, the level of marginalization also influences food insecurity in the surveyed urban and semi-urban communities of Ciudad de Mexico, Estado de Mexico, Oaxaca, and Tamaulipas, as already discussed by other studies [22, 24]. Each factor or the interrelation among different circumstances affects food accessibility and consumption as well as concern about food scarcity.

Evidence from this study demonstrates that individuals who experience food insecurity adopt preventive strategies to confront and resolve food insecurity. Coping strategies become stricter as the worriedness of facing food scarcity in the household materializes. Coping mechanisms are not necessarily more difficult to endure because of restricted food access, as previously discussed [33], but, because some of these strategies resemble the ones utilized in the extreme context of famine [28]. In the study’s case scenario, the individual does not earn sufficient income to buy food in bulks, strategies to cope include hoarding food or prioritizing food expenses, as prior research demonstrates [29, 40]. Households tend to decrease food purchases or stop buying items that are not considered necessities. Hence, the study’s findings reveal that a variety of factors determine food access as well as food quantity, quality, portions, and/or degrees of food starvation.

Thus, a linear path between food access and consumption does not necessarily follow. In contexts that present very high and high levels of marginalization, food insecurity is experienced differently compared to households with medium and lower levels of marginalization. In the latter, issues with accessibility are resolved. Nonetheless, accessibility does not guarantee consumption among individuals who despite being employed in white collar occupations experience a severe decline of purchasing power through the payment cycle due to depressed salaries and high levels of inflation.

Households located in contexts with very high and high levels of marginalization face difficulties with ensuring the supplies needed for a varied diet, considering the issues with food accessibility that characterize semi-urban areas. These geographies tend to be sparsely populated and as a result encounter difficulties with transportation more often [41]. Individuals who live in highly marginalized areas must leave their neighborhoods to access food. Transportation increases food cost, indirectly reducing quality, quantity, and variety as consumers must exchange one element over the other. Issues that pertain to food accessibility are further exacerbated by the participants’ low earnings, which along inflation automatically devalue purchasing power [42, 43].

Consequently, some interviewees report not eating one of the three daily meals, while the other two meals are insufficient and consist of low-quality ingredients and small portions. Modifications on consumption increment the degree of food insecurity [3]. Interviewees must cut back on meal portions and/or the quality of the ingredients to prepare meals. Food portions must also be shared with other household members. Implemented strategies at this level of marginalization reflect the methods utilized in context of famine [28]. The situation is aggravated when children, senior adults, and/or ill family members live in the household. Daily family dynamics incorporate tactics to prioritize family members who could experience major nutritional risk with the lack of food as other investigations have shown [26]. Our findings parallel literature that examines highly marginalized environments [24, 27], where people face food insecurity regularly [44.

Concerning contexts with medium levels of marginalization, interviewees identify venues to buy food in their neighborhoods. As in previous categories, the most prominent issue is the limited income to purchase food. Interviewees from this category must not inevitably skip any of the three daily meals. Nonetheless, a few participants have had to implement a course of action to guarantee food for all the family such as lowering food quality and/or reducing meal portions.

At low and very low levels of marginalization, participants implement strategies that resemble the tactics employed by individuals from higher marginal contexts. Our findings indicate that food access does not represent an issue. It is the low income that participants earned that is the subject of concern. Reduced purchasing power through the *quincena* cycle deteriorates the quality of food supplies. In this context, the payment cycle determines food consumption. At the end of the *quincena*, a food basket of high quality is not affordable whereas at the beginning of the *quincena* sufficient food can be purchased. As the end of the *quincena* approaches, individuals must wait until the next payday to purchase food. The study’s participants recognize that financial difficulties affect food consumption even though their living context present high IMM levels. Although meals are not skipped, to make ends meet, portions must be reduced. The tactic illustrates one of the most relevant coping strategies that stems from the fear of running out of food.

The *quincena*, a factor that relates to a specific cultural context, reflects how feelings of concern are experienced across the changing purchasing power of interviewees in a month- span. Feelings of concern increase as consumption capacities decline. This implies—when food variety changes—that food portions are diminished and meals are omitted, which propels a perception and fear of not being able to meet a household’s food needs for a specific number of days. Therefore, days before payday, evidence indicates that food insecurity increases. In this sense, measuring food insecurity in those days can change the result.

Findings from this study also indicate that problems with food consumption generate concern about running out of food. However, different levels of concern must be considered, each level influences households’ decisions and strategies to confront food scarcity. Furthermore, the study’s findings demonstrate that participants are well-aware of the significant increase in food prices. Inflation augments the concern over the uncertainty felt of not being able to purchase food or having to minimize meals’ quantity and quality. Feelings of concern are present among participants from all levels of marginalization. This type of anxious psychological state of mind exacerbates when children are present, and when the breadwinner does not have a stable job is unemployed or ill.

The experience of participants from low and very low levels of marginalization sheds light on the raising precarity of the job market. That is, employed individuals, who work in what are considered professional occupations, might not necessarily have a steady source of cash flow. Considering that an increasing number of jobs might be temporary or flexible in nature (not guaranteeing enough hours per week) [45]. Individuals can also experience unemployment, underemployment, or a high turnover rate. Hence, an employed worker presenting high levels of education can still feel concerned about experiencing food scarcity whenever he/she runs out of money before the *quincena.* This phenomenon might become the future norm if a social policy is not implemented.

The uncertainty triggered by periods of food scarcity has a detrimental psychological impact. Higher levels of stress are associated with the uncertainty that is brought forth by the fear of running out of food [3]. In Mexico, the psychological dimension can very well explain why people who live in contexts where there is greater food insecurity present the highest percentage of individuals who suffer from being overweight, morbid obese, diabetic, and hypertensive—specifically, women and children [46, 47].

At this point, it is important to acknowledge the limitations of this study. Due to the low number of interviews administered to individuals who live in high and very high levels of marginalization, the semi-structure interviews from these categories must be pooled for analysis. Furthermore, the study’s qualitative approach sets limits to generalize from our results. Despite these limitations, analytical themes are saturated, which allowed us to find a link across analytical categories that offer confidence and consistency to the results.

## Conclusions

Based on different levels of marginalization, the instruments to measure food insecurity must be recalibrated to account for the change in food consumption considering the changing patterns of purchasing power through the payment cycle. Drawing from our sample and data analysis, we conclude that public policies must be drafted to account for the food insecurity that is experienced at the end of the payment cycle, the period when food insecurity increases, particularly in contexts of low and very low levels of marginalization. Social programs can be drafted based on our evidence. For instance, depending on the context of marginalization, a money transfer could be granted to consumers for food or vouchers to be exchanged at a convenience store or *tianguis*.

Our analysis calls for rethinking the scales to measure food insecurity. According to our findings, four conclusions must be drawn. First, the fear of experiencing food scarcity is generalized across levels of marginalization. Uncertainty pushes individuals to develop different strategies—cutting back in consumption and lower food quality—to deal with food insecurity. Furthermore, the inability of individuals to secure adequate supplies for the household negatively impact individuals’ well-being: psychological—increasing levels of anxiety and stress—and physical—increasing levels of obesity or malnutrition—due to food consumption of low-cost and high calories. Although current scales inquire about the concern related to food scarcity, the questions are not able to capture the complexity of this important factor. It is therefore vital to redesign questions related to the feelings of concern over confronting food scarcity to measure more precisely the fear of running out of food or living without food.

Second, although severe levels of food insecurity show the adverse conditions experienced by many people, more studies need to be conducted to determine if descriptions related to food insecurity effectively capture the most adverse episodes of hunger. Otherwise, an additional level of food insecurity to account for this type of experiences must be generated, particularly for countries like Mexico where the endemic conditions of poverty, inequality, as well as the minimum impact of social policies [48, 2] produce constant episodes of hunger. Third, the instruments utilized to measure food insecurity must accurately illustrate whether people can obtain and consume the type of foods needed to maintain a varied diet. Fourth, the redesigned categories must also account for regional differences, as well as specific levels of marginalization. Whereas all levels of marginalization in this study share low income levels as the factor that sets limits to food consumption, problems that are not shared across different levels of marginalization can change the way food insecurity is experienced in a specific context—food insecurity increases at the end of the *quincena* in contexts of low and very low levels of marginalization whereas food insecurity remains constant in areas with high and very high levels of marginalization. Consequently, it is essential to include a different weighting factor—specific to the area under study—that allows for comparison over time to track its evolution.

Overall, this study shows the relevancy of accounting for levels of marginalization in the design of public policy to eradicate food insecurity. Drawing from our findings, food insecurity is linked to the context where it develops. Governmental efforts must have a territorial (local) and regional component.

## Data Availability

Datasets used and analyzed in this study are available on reasonable request

## List of abbreviations

CONEVAL: National Council for the Evaluation of Social Development Policy
ELCSA: Latin American and Caribbean Scale of Food Security
EMSA: Mexican Scale of Food Insecurity
FAO: Food and Agriculture Organization
FIES: Food Insecurity Experience Scale
IMM: Municipal Marginalization Index
SDG2: UN Sustainable Development Goals 2
